# DRAGen in Application—An Approach for Microstructural Fatigue Predictions of Non-Oriented Electrical Steel Sheets

**DOI:** 10.3390/ma17112678

**Published:** 2024-06-01

**Authors:** Manuel Henrich, Sebastian Münstermann

**Affiliations:** Institute of Metal Forming, RWTH Aachen University, Intzestraße 10, 52072 Aachen, Germany; sebastian.muenstermann@ibf.rwth-aachen.de

**Keywords:** Representative Volume Elements, microstructure, fatigue, DRAGen

## Abstract

This study investigates multiple cyclic loading scenarios of non-oriented electrical steel sheets through both experimental and numerical approaches. The numerical simulations were conducted using Representative Volume Elements generated with DRAGen. DRAGen allowed for the generation of Representative Volume Elements with a non-cubic shape to cover the complete sheet thickness and enough grains to represent the material’s texture. The experimental results, on the other hand, are utilized to calibrate and validate a prediction model, highlighting the significance of accumulated plastic slip as a suitable parameter correlated with fatigue life. Using the accumulated plastic slip from the simulations, a fatigue fracture locus is introduced, which describes a 3D surface dependent on the maximum stress, fatigue life, and the fatigue stress ratio. The study shows reliable results for the fatigue life prediction using the calibrated fatigue fracture locus. While substantial progress has been made in predicting the fatigue life at multiple fatigue stress ratios, notable disparities between experimental and simulation results suggest the need for further investigations regarding the influence of the surface quality. This observation motivates ongoing research efforts aimed at refining simulation methodologies to better incorporate surface roughness effects. In summary, this study presents a validated model for predicting fatigue life in non-oriented electrical steel sheets, offering valuable insights into material behavior at different loading scenarios and informing future research directions for enhanced structural performance and durability.

## 1. Introduction

To effectively model fatigue failure, it is crucial that the selected methodologies accurately emulate the fundamental physical processes at play. High Cycle Fatigue (HCF) denotes material failure under cyclic loading below its yield strength. However, at the microscopic level, plasticity effects occurring within a few grain diameters can locally compromise the material’s integrity. This phenomenon, as noted by Bauschinger [1], is attributed to the accumulation of dislocations under reverse loading, leading to an irreversible increase in local plasticity.

Studies, such as those conducted by Polák et al. [2,3,4] utilizing Scanning Electron Microscopy (SEM), have revealed that fatigue crack initiation predominantly occurs at Persistent Slip Bands (PSBs) within steel microstructures. PSBs arise due to the aggregation of dislocations within grains possessing favorable crystallographic orientations under cyclic loading conditions. Consequently, short cracks tend to initiate at these sites. Also, their subsequent propagation is influenced by various microstructural attributes, including grain boundaries, grain orientation, and the presence of inclusions.

Furthermore, it is noteworthy that Crystal Plasticity (CP) models, in combination with Finite Element Method (FEM) representations of microstructures, have emerged as preferred tools for simulating fatigue failure. These models allow for a detailed examination of the interaction between different microstructural features and loading conditions, providing valuable insights into the fatigue behavior of materials. Thus, combining experimental observations with computational simulations offers a comprehensive understanding of fatigue failure mechanisms and aids in developing more robust materials and structures [5].

Multiple studies show that numerical simulations in combination with microstructure models have a high potential for predicting fatigue properties of metallic materials. One noteworthy approach for predicting these properties lies in the statistical analysis of strain fields from multiple simulations shown by Mughrabi [6], Sharaf et al. [7], Cruzado et al. [8] and Shahmardani and Hartmaier [9]. These studies utilize the statistical nature of the Representative Volume Elements (RVEs) as a linking element between simulation and experimental results.

Yet, the RVEs used in these and other studies solely rely on grain-dependent parameters such as the grain size and shape [10]. These parameters are not always sufficient to describe materials in their overall complexity. Henrich et al. [11] introduced an RVE generator called Discrete RVE Automation and Generation (DRAGen), which covers more than just grain-dependent parameters. One of these features is the shape of the RVE itself. DRAGen allows users to generate RVEs that have different dimensions in each direction. This is especially useful for thin metal sheets such as electrical steels. The deep understanding of mechanical mechanisms in electrical steels is especially interesting due to the increasing importance of electrical motors due to the challenge to combat climate change [12,13,14,15].

Electrical steels among other things are characterized by their high silicon content, reaching up to 4.5%. This causes a change in dislocation movement. Screw dislocations have a lower tendency towards cross-slip, and the yield strength is increased due to solid solution hardening. Collectively, these factors significantly influence the fatigue life by reducing the appearance of PSBs. leading to fatigue-resistant scenarios far in the plastic regime. In other words, the maximum fatigue stress $\sigma_{max}$ must surpass the yield stress to induce material failure due to fatigue [16,17].

Traditional fracture mechanics approaches, e.g., using the J-Integral, depend on a predefined crack. The results of these approaches are directly linked to the crack shape, meaning the crack itself is to be seen as a material parameter [18]. In this scenario, the microstructure influence and the crack’s influence on the mechanical response of the material cannot be isolated anymore, and the correct calibration of the assumed crack is near impossible. Therefore, efforts were made to describe the crack incubation phase based on a crack-free microstructure [6,8,10]. The study carried out by Gillner and Münstermann [10] argues that the accumulated plastic strain $p_{acc}$ as introduced by Dunne et al. [19] can be used for the determination of indicators for fatigue failure because it describes the statistical characteristics of the PSB initiation and the statistical formation of short cracks on a phenomenological basis. Statistical formation of short cracks in this context means the statistical description of stress hot spots serving as initiation sites for short cracks. Although these simulation approaches describe the statistical behavior of short crack incubation, cracks are not physically considered. Fracture mechanics approaches, e.g., as presented by Zhu et al. [20], assume a physically existing crack. Since it is a major difficulty to calibrate the pre-existing cracks in advance correctly, the present study aims to perform a fatigue life estimation for the crack-free material. Considering this objective, the work presented by Gillner and Münstermann [10] seems to be a perfect match for the fatigue life prediction of electrical steels. However, in Gillner and Münstermann [10] as well as Sharaf et al. [7], microstructural short cracks are assumed to surpass a few grains in size. Since the electrical steel investigated in this study has large grains in combination with a small sheet thickness of 300 μm, this definition for short cracks is already considered a critical crack causing sudden failure. Especially in the work of Gillner and Münstermann [10], the attempt was made to predict the fatigue life for multiple fatigue stress ratios R=σminσmax, where $\sigma_{min}$ is the minimal stress value within one cycle, and $\sigma_{max}$ is the maximal stress value. This approach is based on a calibrated set of parameters, an estimation for crack incubation, and a second estimation for crack growth. The parameters are used in an extrapolation term that is added to the number of cycles for crack incubation and represents the crack growth phase. This extrapolation term takes the statistical nature of the material into account through the statistics covered in the RVEs. However, since the cracks that are considered short cracks in the work presented by Gillner and Münstermann [10] are critical in electrical steels, the authors of this study found that the approach for predicting cyclic behavior as suggested by Gillner and Münstermann [10] does not lead to reliable results, especially when varying the fatigue stress ratio *R*.

Therefore, an approach is presented that relies on the incubation phase only. For this purpose, 3D RVEs was used, generated with DRAGen. Also, the geometrical feature of non-cubic RVEs implemented in DRAGen was used. This feature ensures the correct surface grain-to-grain ratio, which describes the ratio between the number of grains located on the sheet surfaces and the total number of grains. This ratio is higher in electrical steel than in common steel due to the large grain size. The higher ratio correlates with a greater notch sensitivity, causing a broad scatter in fatigue failure. Also, the failure tends to start at the surface rather than the inside of the material. Also, using this feature ensured a reasonable amount of grains in the RVEs and a good representation of the texture. The generated RVEs were used for cyclic simulations, and the accumulated plastic strain was extracted just like in the study presented by Gillner and Münstermann [10]. However, in the post-processing step, a new approach is developed to describe the fatigue stress ratio dependency, which does not rely on a second extrapolation term. The goal is to calculate the fatigue life directly from each RVE simulation. The calculation-presented life is similar to the approach presented by Sayer et al. [21]. Yet, in contrast to these two studies, the critical Fatigue Indicator Parameter ($FIP_{crit}$) was not assumed to be constant for different fatigue stress ratios. This leads to the following two hypotheses addressed in this study:

- The mean maximum accumulated plastic strain, which is treated as Fatigue Indicator Parameter (FIP) in this study, follows a relationship that is dependent on the fatigue stress ratio *R* and can be obtained empirically.
- The fatigue life can be calculated directly from the accumulated plastic slip once the $FIP_{crit}$ relationship is known.

Also, by finding the correct $FIP_{crit}$ relationships, fatigue life can be calculated directly from the accumulated plastic slip.

The methodologies employed in this study are outlined as follows: the material characterization described in Section 2 is initially conducted, followed by a brief description of the CP model in Section 3 that is utilized. Subsequently, the methodology for predicting the fatigue life is presented in Section 4. The results of the simulations are then presented in Section 5, and Section 6 concludes the paper by synthesizing the findings from the study.

## 2. Material Characterization and Microstructure Reconstruction

The material used in this study is a NO30 electrical steel grade with a thickness of 300 μm. The material has already been characterized in the publication by Henrich et al. [11]. Accordingly, the present study utilizes and briefly summarizes the data from this prior publication. The figures presented herein are based on datasets originally developed for Henrich et al. [11], yielding graphs that replicate the results. These figures are included in the appendix; for detailed methodologies concerning the data acquisition and analysis techniques used in these figures, readers are referred to the cited publication.

**Figure A1:**
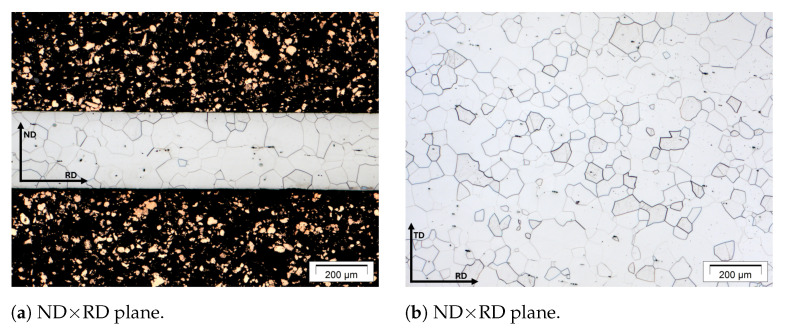
LOM image of nital etched microstructure of cross sections in different directions.

**Figure A2:**
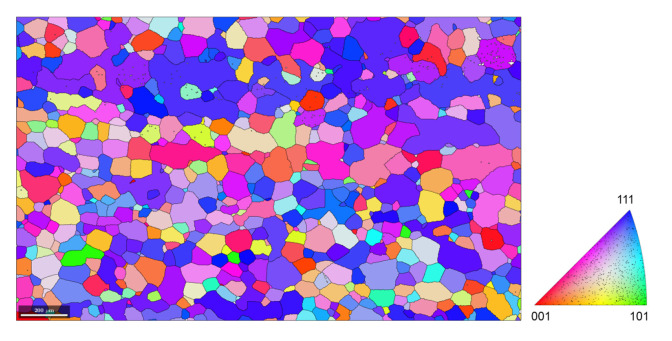
EBSD grain map showing large angle grain boundaries at $5^{{}^{\circ}}$ and bigger.

**Figure A3:**
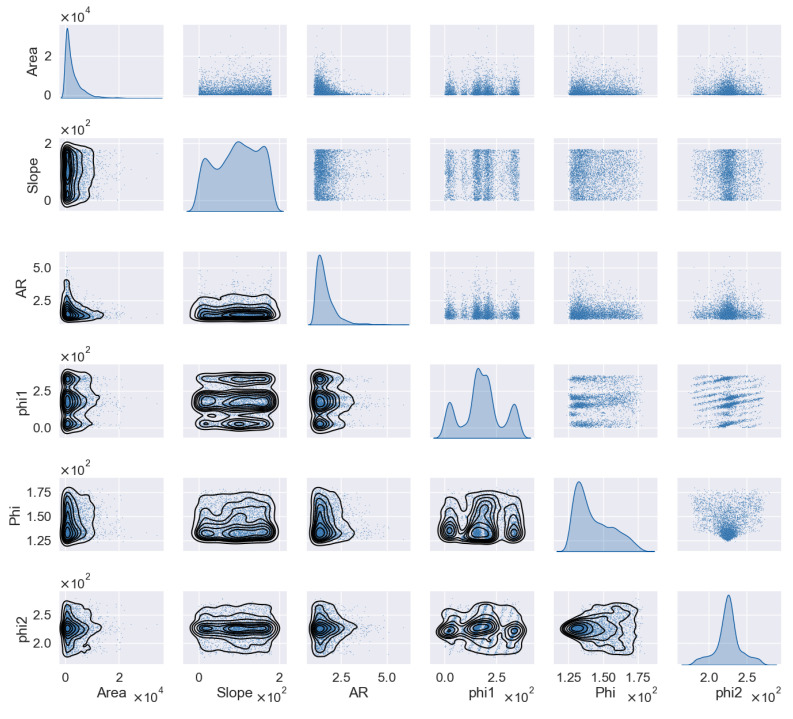
Statistical grain data from EBSD grain map.

**Figure A4:**
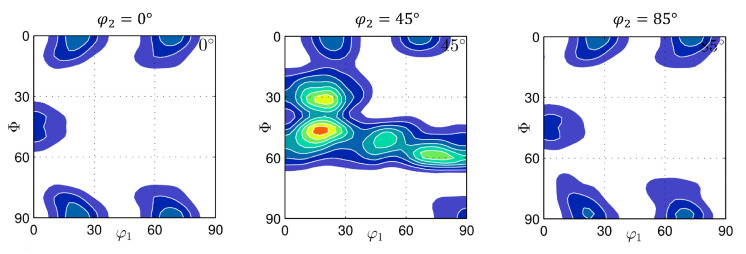
XRD measurement shown in section plot.

For the accurate microstructure reconstruction, the material was analyzed with Light Optical Microscopy (LOM) and with Electron Backscatter Diffraction (EBSD), and for the mechanical properties, tensile tests were performed. The LOM images were used for visual validation of the EBSD measurements. All information for further processing was extracted from the EBSD data. For the grain reconstruction in the EBSD data, a threshold misorientation angle of 5° was applied to meet a visual agreement with the LOM images. The grain reconstruction was performed with the MATLAB toolbox MTEX (version 5.10.2) [22]. Additionally to the EBSD measurement, an X-ray Diffraction (XRD) measurement for texture analysis was performed. The results of all microstructure measurements are shown in Figure A1, Figure A2, Figure A3 and Figure A4.

**Figure 1:**
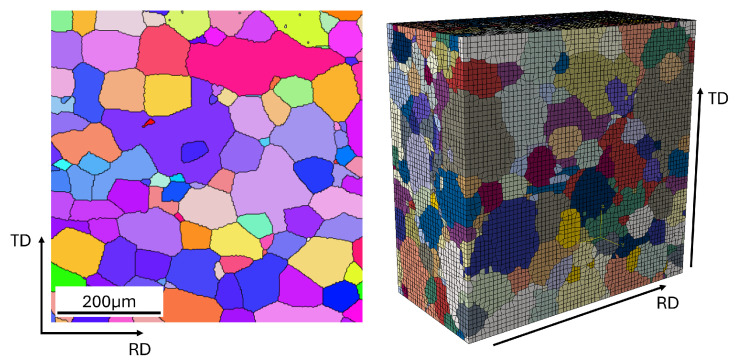
Comparison of EBSD (**left**) and RVE (**right**) generated with DRAGen.

**Figure A5:**
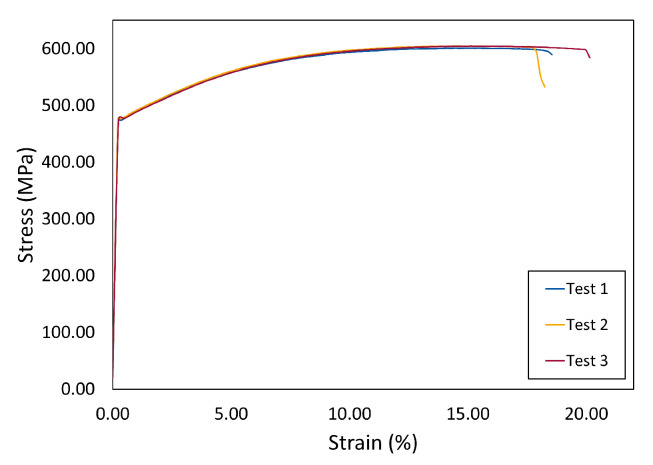
Technical stress strain curves measured in a quasi-static tensile test.

**Figure A6:**
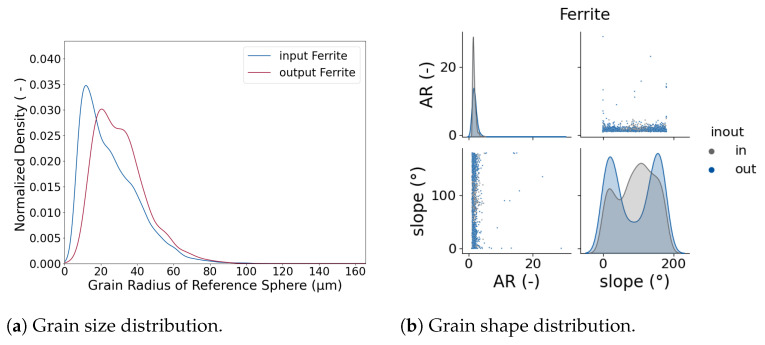
Comparison of RVE input and output for grain size and shape distributions.

**Figure A7:**
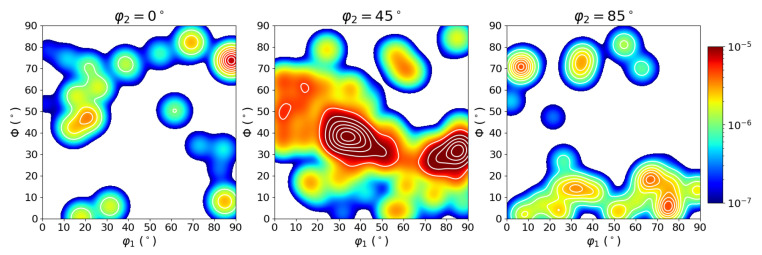
Section plot extracted from one arbitrarily chosen RVE.

Figure A1a,b shows the grains in two different planes: the Rolling Direction (RD) vs. Normal Direction (ND) plane and the RD vs. Transverse Direction (TD) plane. The grains appear coarse with only a few grains covering the complete sheet thickness. For both planes, it is also visible that there is no preferred direction for the grain shape. Therefore, the grain shapes can be assumed to be spheres. Since spheres are of the same dimension in each direction, only one EBSD image is needed for this material. Hence, the EBSD measurement was performed on the RD × ND plane to collect as many grains as possible. This EBSD analysis leads to multiple grain maps like the one shown in Figure A2. The statistical data extracted from all the maps, including the one in Figure A2, is shown in Figure A3. This complete dataset was used for further processing with DRAGen. The XRD measurement for the texture analysis is depicted in the section plot shown in Figure A4. The XRD data are used for validation purposes for the synthetic texture in the RVEs generated with DRAGen. The tensile test data were used for calibration purposes for the CP model and are shown in Figure A5. Using the statistical data from these characterization methods as input for the RVE generator DRAGen, multiple RVEs could be generated. The generation process in DRAGen runs fully automatic and is based on discrete data processing algorithms that randomly fill the RVE volume with grains. In the following, the core logic of DRAGen is briefly explained. For a more detailed description of the tool, the two publications by Henrich et al. [11,23] are recommended. DRAGen is built upon three main parts, a Wasserstein Generative Adverserial Network (WGAN) supported input generator, a discrete Random Sequential Addition (RSA) algorithm, and a discrete tesselation algorithm. Once trained, the input generator can reconstruct synthetic grain information as it can be extracted from EBSD measurements. This includes multidimensional and interdependent statistical distributions of microstructure features. The discrete RSA uses this input data generated with the WGAN and places ellipsoids as seeds in a discrete volume. The ellipsoids have an assigned crystallographic orientation, and they will represent 40% of the grain volume at the end of the reconstruction. This is necessary for the correct and efficient placement of the ellipsoid. After all ellipsoids were placed in the volume, a discrete growth, also referred to as the tesselation algorithm, starts. This tesselation algorithm has an implemented volume control to maintain the correct grain size distribution. Once the empty volume between the ellipsoids has been filled completely due to the tesselation, the RVE is transformed into an Finite Element (FE) mesh and an Abaqus input file is generated. Next to the microstructure features linked to the grain structure, other features are also available in DRAGen. One of these features is the dimensionality of the RVE itself. DRAGen allows the generation of non-cubic RVEs, which is inevitable for the representative reconstruction of thin electric steel sheets. These RVEs were the basis for simulating different cyclic loading cases in the present study. Exemplary data for the microstructure reconstruction taken from an arbitrarily picked RVE is shown in Figure A6 and Figure A7. In these figures, the experimental data (input) agree well with the synthetic data coming from the RVE (output). Thus, the reconstruction was properly carried out. Also, a visual comparison as shown in Figure 1 demonstrates the accuracy of the reconstruction mechanisms underlying the RVE, as the reconstructed microstructure shows a very similar grain structure as seen in the EBSD image.

## 3. Crystal Plasticity Model

The material model used in combination with DRAGenRVEs is a phenomenological CP model that describes the mechanical behavior of a single crystal. The used model depends on the crystallographic orientations and does not incorporate grain boundary effects besides the crystallographic misorientation defined by the Euler angles for each grain. The model is calibrated with the macroscopic tensile tests shown in Figure A5. Therefore, inter-granular mechanical effects are covered through the calibration approach and the material’s hardening behavior. In the following, the most basic functions of the CP model are shown. These functions contain the parameters that must be calibrated for each material. First the deformation gradient F is decomposed into an elastic and a plastic component $F_{e}$ and $F_{p}$ [24,25]:

(1) $$\begin{array}{c} F=F_{e}F_{p} \end{array}$$

The rate of the plastic component is then calculated using the plastic velocity gradient:

(2) $$\begin{array}{c} {\dot{F}}_{p}=L_{p}F_{p} \end{array}$$

while $L_{p}$ is calculated from the shear rate ${\dot{\gamma }}^{\alpha }$, the according slip direction $m^{\alpha }$ and normal vectors $n^{\alpha }$ of the slip system α:

(3) $$\begin{array}{c} L_{p}=\sum_{\alpha =1}^{n}{\dot{\gamma }}^{\alpha }m^{\alpha }⊗n^{\alpha } \end{array}$$

The shear rate ${\dot{\gamma }}^{\alpha }$ is a function of the resolved shear stress $\tau_{eff}^{\alpha }$ and the critical resolved shear stress $\tau_{c}^{\alpha }$ [26]:

(4) $$\begin{array}{c} {\dot{\gamma }}^{\alpha }={\dot{\gamma }}_{0}\cdot {\left|\frac{\tau_{eff}^{\alpha }}{\tau_{c}^{\alpha }}\right|}^{\frac{1}{m}}\cdot sign\left(\tau_{eff}^{\alpha }\right) \end{array}$$

${\dot{\gamma }}_{0}$ and *m* are material-related parameters representing the reference shear rate and the slip rate sensitivity, while α indicates the current slip system, and $\tau_{c}$ the critical resolved shear stress. $\tau_{eff}^{\alpha }$ is the effective resolved shear stress resulting from the resolved shear stress $\tau^{\alpha }$ and the kinematic back stress $\chi^{\alpha }$. The expression for the back stress $\chi^{\alpha }$ was originally proposed by Frederick and Armstrong [27] and is implemented as shown below as well as the formulation of $\tau^{\alpha }$ according to Schmidt’s law:

(5)τeffα=τα−χα(6)τα=S·mα⊗nα(7)χ˙α=C·γ˙α−D·γ˙αχα

S represents the second Piola–Kirchoff stress.

The critical resolved shear stress is calculated as follows [28]:

(8) $$\begin{array}{c} \tau_{c}^{\alpha }=\tau_{0}+\int_{0}^{t}q_{\alpha \beta }\left[h_{0}{\left(1-\frac{\tau_{c}^{\beta }}{\tau_{s}}\right)}^{a}\right]\left|{\dot{\gamma }}^{\beta }\right|dt \end{array}$$

In Equation (8), $\tau_{0}$ represents the initial critical resolved shear stress and $q_{\alpha \beta }$ the latent hardening matrix. The parameters $h_{0}$, $\tau_{s}$ and *a* are material parameters that need to be fitted during the calibration process, as well as the parameters *C*, *D*, ${\dot{\gamma }}_{0}$ and *m*. Also, $\tau_{0}$ is fitted during the calibration process while $q_{\alpha \beta }$ is defined as 1.0 for co-planar and 1.4 for non-coplanar slip systems α and β [29]. In addition to the critical shear stress calculation, the crystallographic orientations of the grains are updated. This updating step is based on the polar decomposition of the elastic part of the deformation gradient $F_{e}$ shown below [24]:

(9) $$\begin{array}{c} F_{e}=R\cdot U \end{array}$$

In this decomposition, R represents the rotation matrix describing the rotational component of the material deformation and U is the matrix describing the translational part. The rotation matrix R is used to update the Euler angles in each simulation step. A substantial summary of the crystal plasticity theory and the mechanisms behind the theory can also be found in the work published by Roters et al. [30] as well as a summary of the presented formulations.

The material model is implemented in an Abaqus UMAT user subroutine and depends on the crystallographic orientation defined at each integration point. DRAGen defines these crystallographic orientations for each grain during the generation process. Afterwards, these orientations are passed to a unique abaqus user material definition for each grain. Besides these orientations coming from DRAGen, the user material considers the material parameters of the CP model. For all simulations in the presented work, Abaqus 2022 was used.

**Figure 2:**
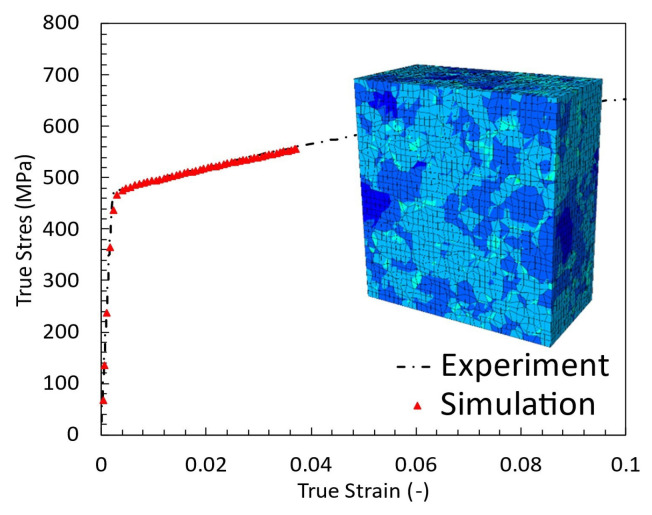
Comparison of experimental and simulation stress-strain data.

The calibration of the CP parameters was carried out iteratively with the tensile tests shown in Figure A5. At the beginning of the calibration process, an initial set of parameter values based on the existing literature as published by, e.g., Gillner and Münstermann [10], Tasan et al. [31] or Cruzado et al. [8], previous experience or theoretical estimates were used. With these initial parameters, the crystal plasticity simulations were performed, and the results were compared with the experimental data. Discrepancies between the simulation results and the experimental data were addressed by manually adjusting the parameters. Attention was paid to how the changes affected the response of the model. This process was repeated iteratively until the simulation results matched the experimental data sufficiently. Parameter convergence was considered achieved when further adjustments led to negligible improvements in the agreement between the simulation and experimental results. The calibration result is shown in Figure 2 and the resulting parameters in Table 1. The kinematic hardening parameters *C* and *D* were neglected in this study and set to C=D=0 since all experiments were only conducted under tensile pulsating loads, and there is no reverse in the load which could significantly cause the effect described by Bauschinger [1]. The reason for this choice of loading scenario is directly linked to the materials’ dimension, which makes it impossible to apply any negative loads to the samples because the sheet material is too thin to prevent it from buckling under compressive loads.

## 4. Fatigue Life Prediction

The loading conditions in the simulations on the non-cubic RVEs were applied to the non-quadratic RVE surfaces only. By that, the quadratic, front, and back surfaces of the RVE see the same loading condition as the surface in the experiment, leading to the correct mechanical response. The predicting method of fatigue life in this study is based on the assumption that the fatigue life in electrical steels is limited by the material’s resistance against irreversible accumulated plasticity. Thus, in each simulation, the accumulation of plastic slip $p_{acc}$ is calculated from plastic velocity gradient $L_{p}$ representing the sum of the dislocation slips on all slip systems as shown in Equation (2). The calculation is performed on every integration point by the CP subroutine as follows [19]:

(10) $$\begin{array}{c} p_{acc}=\int_{0}^{t}\sqrt{\frac{2}{3}\left(L_{p}:L_{p}\right)}dt \end{array}$$

From this accumulated plastic slip (also referred to as accumulated plastic strain), the maximum grain volume-weighted average of all grains can be calculated by

(11) $$\begin{array}{c} P_{mps}=\max\left(\frac{1}{V_{gr}}\sum_{i=1}^{N_{E}^{gr}}p_{acc,i}V_{i}^{gr}\right) \end{array}$$

where $V^{gr}$ is the grain volume, $V_{i}^{gr}$ is the element volume, and $N_{E}^{gr}$ is the number of elements for the grain. Since only the maximum is considered, one $P_{mps}$ value is obtained for each simulation. It is extracted after the last step of each simulation. According to the study by Gillner and Münstermann [10] the evolution of $P_{mps}$ during one simulation can be considered linear. The study showed this is also true for very long simulations covering over 100 cycles. Using this knowledge, it is feasible to simulate only a small number of cycles and calculate the average of $P_{mps}$ over the simulated number of cycles $N_{cycles}$. In the presented study, six cycles were simulated as proposed by Gillner and Münstermann [10]. The average value $\Delta P_{mps,i}$ represents the maximum grain averaged accumulated plastic slip, which is added during one cycle to the material in the RVE with the index *i* with $i=1...N_{simulations}$ and $N_{simulations}$ being the total number of simulations at the same loading scenario. The calculation of $\Delta P_{mps,i}$ is shown below:

(12)ΔPmps,i=Pmps,iNcycles(13) FIP=1Nsimulations∑i=1NsimulationsΔPmps,i

In Equation (13), the average of $\Delta P_{mps}$ across all RVE simulation with the same loading scenario is calculated and considered the FIP for this study. Due to its origin in the deformation gradient F this FIP considers the crystallographic orientation, as well as the present stress and strain.

Parallel to the simulations, cyclic tests were carried out at different loading amplitudes while each loading amplitude was covered with multiple tests at equal conditions. These experiments were conducted using flat tensile specimens of the geometry A50 according to DIN/EN ISO 6892-1 [32]. The testing machine used was a servo-hydraulic machine, and the test frequency was 25 Hz. The experiments led to multiple sets of numbers of cycles until fracture under a certain loading amplitude and R-value. The loading scenarios used for the experiments are also used as loading conditions in the simulations. That way, $\Delta P_{mps,i}$ can be correlated with the experimentally estimated fatigue life, as has been shown in the publication by Sayer et al. [21]. The mean value for the number of cycles at each loading scenario ${\bar{N}}_{exp}\left(\sigma_{max},R\right)$ is then used for the determination of the critical $FIP_{crit}\left(\sigma_{max},R\right)$ as shown below:

(14) $$\begin{array}{c} FIP_{crit}\left(\sigma_{max},R\right)=FIP\cdot {\bar{N}}_{exp}\left(\sigma_{max},R\right) \end{array}$$

As shown in Equation (14), $FIP_{crit}\left(\sigma ,R\right)$ is dependent not only on the stress amplitude $\sigma_{a}$ but also on the R-value. Therefore, it represents a surface in 3D space that can be calibrated by experiments and simulations performed at the same coordinates. This surface is called Fatigue Fracture Locus (FFL) in the following. Inspired by the Basquin Equation [33] and an extension for an R-value dependency, the mathematical relationship for the FFL is postulated as shown below:

(15) $$\begin{array}{c} FIP_{crit}=e^{A}\cdot {\left(\sigma_{max}\cdot \left(1-R\right)\right)}^{b} \end{array}$$

Herein, the parameters *A* and *b* are fitting parameters that need to be calibrated by estimating the $FIP_{crit}$ according to Equation (14) by combining simulation and experimental results and performing a curve fit afterwards.

The experimental data used for calibration are listed in Table 2.

Once the FFL is calibrated, the fatigue life for any stress amplitude and R-value combination within the valid bounds of calibration can be predicted using the according FIP from the simulation by rearranging Equation (14) to *N* as shown in Equation (16) and to calculate the fatigue life for each single RVE insert $\Delta P_{mps,i}$ as shown in Equation (17):

(16) $$\begin{array}{cc} {\bar{N}}_{pred}\left(\sigma_{max},R\right) & =\frac{FIP_{crit}\left(\sigma_{max},R\right)}{FIP} \end{array}$$

(17) $$\begin{array}{cc} N_{pred,i}\left(\sigma_{max},R\right) & =\frac{FIP_{crit}\left(\sigma_{max},R\right)}{\Delta P_{mps,i}} \end{array}$$

## 5. Simulation Results

For the calibration of the FFL, the loading conditions from Table 2 were applied to RVE simulations. The same 25 RVEs were used for each loading scenario. From each of these 25 RVEs, the FIP was extracted, and the mean of all 25 FIP was taken. This mean FIP was then multiplied with ${\bar{N}}_{exp}\left(\sigma_{max},R\right)$ in Table 2. That way the table is extended by two more columns as shown in Table 3.

**Figure 3:**
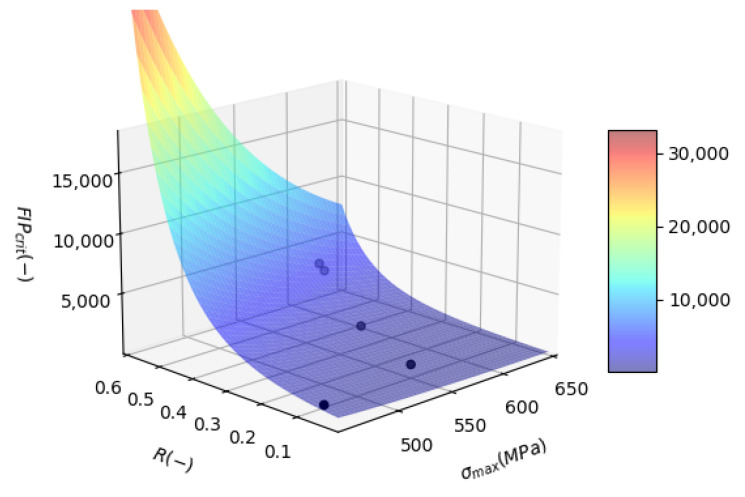
Calibrated FFL, showing the surface in color and the experimental data used for calibration with black markers. Calibrated parameters: A=32.32 and b=−4.20.

The values from the three columns *R*, $\sigma_{max}$ and $FIP_{crit}$ are used to calibrate the FFL surface using a curve fitting method and Equation (15). The resulting surface with parameters A=32.32 and b=−4.20 is shown in Figure 3. The perceived initial arbitrariness in the contour of the surface plot for the $FIP_{crit}$ can be clarified on closer inspection. Notably, $FIP_{crit}$ values increase substantially under conditions of low maximum stress coupled with high fatigue stress ratios *R*. This behavior can be elucidated by considering high *R* values, which mimic the loading conditions of a tensile test. Simultaneously, the maximum stress in this scenario remains about 100 MPa below the material’s Ultimate Tensile Strength (UTS), suggesting that failure due to this loading regime is unlikely. However, the simulated FIP cannot decrease to the minimal levels necessary if $FIP_{crit}$ was to be treated as a constant value, given that the material state is fully plastic. Consequently, the FFL must be adjusted to reflect this influence, resulting in a pronounced rise in the FFL under such conditions.

Conversely, under conditions of low *R* and high maximum stress values, the resultant high FIP values from simulations align well with the observed finite fatigue lives; thus, $FIP_{crit}$ is observed to take on lower values. This inverse relationship between $FIP_{crit}$ and the stress conditions highlights the need for a dynamic adaptation of $FIP_{crit}$ to accurately represent the material behavior under varying fatigue-loading scenarios. This understanding helps refine predictive models for fatigue life based on FIP models.

Using the calibrated FFL and a second set of simulations, it is demonstrated how the fatigue life prediction can be applied. The loading scenarios for the fatigue life predictions are shown in Table 4.

**Figure 4:**
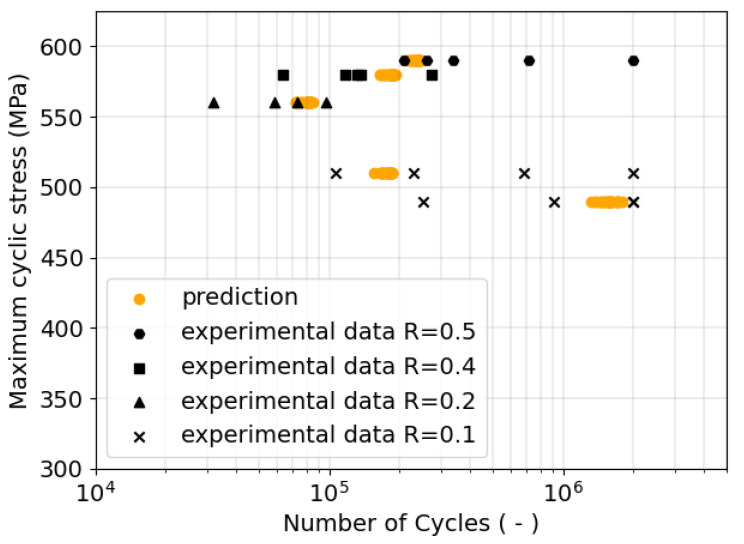
Predicted SN data (orange circles) compared with experimental SN data (black symbols) at different R-values. Each orange circle represents one RVE simulation and each black marker one experiment.

In the prediction step for each RVE, a unique lifetime is calculated based on $FIP_{crit}$ calculated with Equation (14). Experiments with the same loading scenarios were carried out for validation purposes. The predicted values along with the experimental results for validation are shown in Figure 4.

The comparison of predicted experimental values in Figure 4 clearly shows that all predicted lifetimes for the RVE simulations lie within the experimental scatter and are, therefore in good agreement with the validation data. The calibrated FFL can thus be used for the fatigue life prediction within the valid bounds of the calibration scenarios. This result proves that the hypothesis formulated in the beginning is true for this material, and a variable formulation of the FFL across multiple fatigue stress ratios does lead to reliable fatigue life predictions.

However, as depicted in Figure 4, a notable discrepancy exists between the scatter in the experimental and simulation results. This discrepancy could be attributed to the surface quality of the samples or microstructural homogeneities that were still not perfectly reconstructed. The RVEs used in simulations essentially represent perfectly polished samples free of surface notches, resulting in a scatter solely influenced by the microstructure. Notably, this scatter increases at lower stress levels, with the scatter at $\sigma_{max}=$ 490 MPa exhibiting more pronounced variations than at higher stress levels. In contrast, experimental results exhibit scatter influenced by a combination of microstructure and surface quality. To evaluate, if the scatter in the experimental data is mainly influenced by the surface quality, the microstructure, or a mixture of both, the surface roughness should be considered in the RVEs as well. Using the approach presented here, extrapolating the fatigue life from a simulation of six cycles and analyzing the microstructure’s orientation evolution afterwards would not be meaningful because the experimental microstructure has seen several thousand or even million cycles. Introducing the surface roughness into RVE simulations, on the other hand, does influence the simulation results, as shown by Henrich et al. [34]. Therefore, it would increase the information content of the simulations and allow a conclusion on the origin of the scatter and consequently allow a comparison with the findings in literature [17]. Nonetheless, the findings made in this study show the potential for the approach to assume a fatigue stress ratio dependency in FIPs for reliable fatigue life predictions.

## 6. Conclusions and Outlook

This study has successfully developed and validated a model for predicting the fatigue life of non-oriented electrical steel sheets, employing microstructure simulations to capture fatigue behavior within established calibration bounds. In the presented study, multiple cyclic loading scenarios were investigated both experimentally and numerically. The experimental data, categorized into two groups, served to calibrate and validate the prediction model. The results confirm that the accumulated plastic slip is a reliable predictor of fatigue life in electrical steels, and the presented simulation approach utilizing RVEs leads to reliable fatigue life predictions throughout different fatigue stress ratios.

Furthermore, the findings validate the assumption that fatigue crack incubation significantly influences fatigue life, confirming the postulated equation for the FFL with the presented material. The initial hypothesis that the $FIP_{crit}$ should be variable, not constant, has been validated, suggesting a paradigm shift in fatigue life prediction methodologies that should be pursued in subsequent research. This could lead to the validation of the equation across diverse materials, potentially uncovering a universal law governing the FFL.

While the proposed model aligns well with theoretical expectations, it also reveals discrepancies in the level of scatter compared to experimental values, presumably highlighting the influence of surface quality. This discrepancy underscores the need for further exploration of the interplay between surface roughness and microstructural effects in fatigue behavior. For a precise validation of the assumption that surface quality is the major reason for the experimental scatter, a subsequent study is planned using RVEs considering the surface quality. Also, it is recommended for future studies to adjust microstructure models to include surface roughness profiles, as preliminary evidence indicates these models’ sensitivity to surface quality [34]. Such advancements could pave the way for more accurate predictive models, enhancing the current understanding of material behavior under cyclic loading conditions.

## Figures and Tables

**Table 1:** CP-Parameters for electrical steel grad calibrated for this study.

${\dot{\gamma }}_{0}$ (1/s)	*m* (-)	$h_{0}$ (MPa)	$\tau_{s}$ (MPa)	a (-)	$\tau_{0}$ (MPa)	C	D
0.001	0.0016	1000	750	2.4	250	0	0

**Table 2:** Experimental results from fatigue testing for FFL calibration.

*R* (-)	$\sigma_{\max}$ (MPa)	${\bar{N}}_{\exp}\left(\sigma_{\max},R\right)$ (-)
0.1	470	906,101
0.1	550	104,420
0.3	570	157,915
0.5	595	226,987
0.5	600	177,093

**Table 3:** Experimental results from fatigue testing for FFL calibration and FIP extracted from simulations.

*R* (-)	$\sigma_{\max}$ (MPa)	${\bar{N}}_{\exp}\left(\sigma_{\max},R\right)$ (-)	${\mathrm{FIP}}_{\mathrm{mean}}$	FIP _*crit*_
0.1	470	906,101	0.00014	122.54
0.1	550	104,420	0.00988	1031.46
0.3	570	157,915	0.01105	1745.45
0.5	595	226,987	0.02046	4645.81
0.5	600	177,093	0.02197	3891.16

**Table 4:** Loading scenarios for fatigue life prediction.

*R* (-)	$\sigma_{\max}$ (MPa)
0.1	490
0.1	510
0.2	560
0.4	580
0.5	590

## Data Availability

The raw data supporting the conclusions of this article will be made available by the authors on request.

## Abbreviations

The following abbreviations are used in this manuscript:

CP	Crystal Plasticity
DRAGen	Discrete RVE Automation and Generation
EBSD	Electron Backscatter Diffraction
FE	Finite Element
FEM	Finite Element Method
FIP	Fatigue Indicator Parameter
$FIP_{crit}$	critical Fatigue Indicator Parameter
FFL	Fatigue Fracture Locus
HCF	High Cycle Fatigue
LOM	Light Optical Microscopy
ND	Normal Direction
PSB	Persistent Slip Band
RD	Rolling Direction
RSA	Random Sequential Addition
RVE	Representative Volume Element
SEM	Scanning Electron Microscopy
TD	Transverse Direction
UTS	Ultimate Tensile Strength
WGAN	Wasserstein Generative Adverserial Network
XRD	X-ray Diffraction
